# Long-term therapy of interferon-alpha induced pulmonary arterial hypertension with different PDE-5 inhibitors: a case report

**DOI:** 10.1186/1476-7120-3-26

**Published:** 2005-09-02

**Authors:** Nicoline Jochmann, Felix Kiecker, Adrian C Borges, Maja A Hofmann, Stephan Eddicks, Wolfram Sterry, Gert Baumann, Uwe Trefzer

**Affiliations:** 1 Department of Cardiology, Charité – Universitätsmedizin Berlin, Campus Mitte, Berlin, Germany, Schumannstrasse 20/21, 10117 Berlin, Germany; 2 Department of Dermatology and Allergy, Skin Cancer Centre, Charité – Universitätsmedizin Berlin, Schumannstrasse 20/21, 10117 Berlin, Germany

**Keywords:** pulmonary arterial hypertension, PAH, PDE-5 inhibitor, interferon-alpha

## Abstract

**background:**

Interferon alpha2 is widely used in hepatitis and high-risk melanoma. Interferon-induced pulmonary arterial hypertension as a side effect is rare.

**Case presentation:**

We describe a melanoma patient who developed severe pulmonary arterial hypertension 30 months after initiation of adjuvant interferon alpha2b therapy. Discontinuation of interferon did not improve pulmonary arterial hypertension. This patient could be treated successfully with phosphodiesterase-5 inhibitor therapy.

**Conclusion:**

This is only the 5th case of interferon-induced pulmonary arterial hypertension and the first documented case where pulmonary arterial hypertension was not reversible after termination of interferon alpha2 therapy. If interferon alpha2 treated patients develop respiratory symptoms, pulmonary arterial hypertension should be considered in the differential diagnosis. For these patients phosphodiesterase-5 inhibitors, e.g. sildenafil or vardenafil, could be an effective therapeutic approach.

## Background

The interferons (IFN) are a group of glycoproteins produced by a wide range of cells in response to viruses, mitogens, double-stranded RNA and other substances. They perform immunoregulatory, as well as antiviral and antineoplastic functions, with the latter being the result of inhibition of cell proliferation, enhanced MHC expression and tumor-associated antigen expression. The alpha interferon's (IFN α2a and IFN α2b) act as immunomodulators by enhancing natural killer cells, macrophages and T-lymphocyte function, as well as having antiangiogenic properties. Various forms of IFNs have been evaluated as therapy in a variety of malignant and non-malignant diseases. The major oncologic indications for IFNs include malignant melanoma, renal cell carcinoma (RCC), AIDS-related or HHV-8 associated Kaposi's sarcoma, cutaneous T-cell lymphoma, hairy cell leukemia, and chronic myelogenous leukemia (CML), whereas the non-oncologic indications include viral infections (including hepatitis C and HPV-associated lesions such as condylomata acuminata), multiple sclerosis, keloids, keratoacanthoma, Behcet's disease or hemangioma [1]. IFN α2 is approved in the US and Europe for adjuvant therapy of melanoma and is considered the standard therapy for high-risk melanoma [2]. Among the side effects are flu-like symptoms such as fever, chills and anorexia, myalgia, as well as neuropathies and neuropsychiatric side effects, bone marrow depression, liver and renal failure, heart failure, cardiac arrhythmias, peripheral hypo- and hypertension and vascular side effects like Raynaud's phenomena, digital ulceration and gangrene [2,3]. Pulmonary arterial hypertension (PAH) and interstitial pneumonitis are described as rare side effects [3-8]. We describe a female patient with high risk melanoma who developed severe PAH 30 months after initiation of adjuvant IFN therapy and who could be treated successfully with PDE-5 inhibitor therapy.

## Case Presentation

A 40-year-old woman received excision of a superficial spreading melanoma from the rima ani with a safety margin of 3 cm (Clark-Level IV, tumor thickness 1,82 mm). Lymphatic drainage was detected to both inguinal basins and both excised sentinel lymph nodes were unaffected. None of the staging examinations including computer tomography (CT) of the brain, chest, abdomen and pelvis, as well as lymph node sonography revealed any signs of tumor manifestation. The medical history of the patient was otherwise unremarkable and she was not on any medication. There was no family history of hypertension, heart disease or pulmonary disease. Because of the high-risk nature of the melanoma, the patient started long-term adjuvant therapy with IFN α2b (5 × 10 million U. s.c. per week for 4 weeks followed by 3 × 10 million U. s.c. per week).

**Figure 1:**
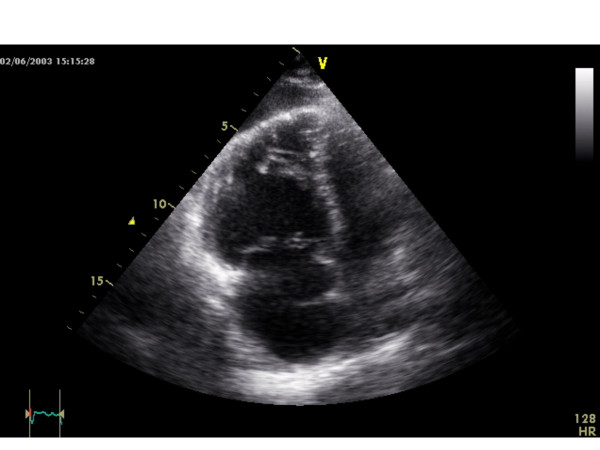
Right ventricular hypertrophy and dilatation at initial investigation with transthoracic echocardiography.

**Figure 2:**
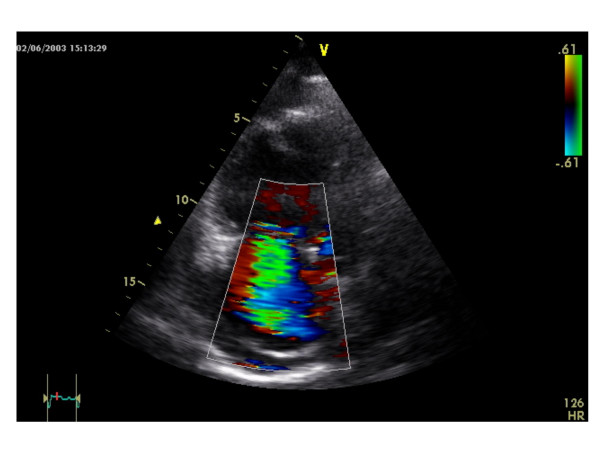
Tricuspid insufficiency grade II–III with a morphological normal valve at initial investigation with transthoracic echocardiography.

After 30 months of IFN α2b treatment the patient reported increasing dyspnea on exertion and afebrile non-productive coughing accompanied by sudden malaise and edema of the lower legs. Electrocardiography showed sinus tachycardia (120 /min) and right axis deviation. A chest x-ray showed signs of right ventricular dilatation and pleural effusion on the right side; no pneumonic infiltrates were seen. Abdominal sonography revealed a significant amount of ascites. The patient was diagnosed with decompensated right heart failure and was therefore hospitalized. Initial investigations with transthoracic echocardiography showed right ventricular hypertrophy and dilatation (Figure 1), PAH with a calculated systolic pulmonary artery pressure (PAP_syst_) of 80 mmHg and tricuspid insufficiency grade II-III with morphologically normal valves (Figure 2), a reduced right ventricular ejection fraction of 40%, a hypokinetic right ventricle and pericardial effusion without signs of tamponade. Laboratory work-up showed slightly increased levels of d-dimers and liver enzymes, while inflammatory markers were within the normal range. There were no signs of vasculitis, hypercoagulability or rheumatologic disorders. A high-resolution CT of the chest revealed no signs of pulmonary embolism, alveolar or interstitial lung diseases, but signs of PAH with a widened central pulmonary artery (40 mm), right ventricular dilatation (> 80 mm), regurgitation of contrast medium into liver veins, a circular pericardial effusion and a 300–400 ml pleural effusion of the right side.

Diagnostic right heart catheter revealed a PAP_mean _of 56 mmHg (PAP_syst _87 mmHg), a pulmonary vascular resistance (PVR) of 1.128 dyn × sec × cm^-5^, an impaired cardiac index and a 3 fold increased total peripheral resistance. Testing of pulmonary vasoreactivity showed a reduction of PAP_mean _from 56 mmHg to 26 mmHg with the PDE-5-inhibitor sildenafil (Table 1).

**Table 1:** Testing of pulmonary vasoreactivity at initial investigation and after six months of PDE-5 inhibitor therapy with right heart catheter

	**HR**	**RR *mmHg syst/diast (mean)***	**PAP *mmHg syst/diast (mean)***	**RA *mmHg***	**PCWP *mmHg***	**CI *l/min/m***^***2***^	**SV *ml/m***^***2***^	**TPR *dyn × sec × cm***^***-5***^	**PVR *dyn × sec × cm***^***-5***^
**baseline**	103	140/89 (104)	87/37 (56)	28	8	1,9	33	3.178	1.128
**baseline ***sildenafil*	96	106/58 (76)	74/14 (26)	13	3	2,2	41	1.290	760
**six months ***sildenafil*	96	105/71 (88)	64/27 (43)	8	4	2,6	46	1.453	708
**six months ***vardenafil*	102	114/69 (83)	63/25 (40)	6	6	2,6	44	1.403	604

baseline = initial investigation; baseline sildenafil = 90 minutes after first dose of 25 mg sildenafil p.o.; six months sildenafil = after 6 months of therapy with sildenafil; six months vardenafil = 120 minutes after first dose of 10 mg vardenafil p.o. HR = heart rate; RR = blood pressure; PAP = pulmonary arterial pressure; RA = right atrial pressure; PCWP = pulmonary capillary wedge pressure; CI = cardiac index; SV = stroke volume; TPR = total peripheral resistance; PVR = pulmonary vascular resistance.

Therefore, treatment with 3 × 25 mg sildenafil per day was initiated. No side effects occurred. After one month, tricuspid insufficiency improved to grade I-II. After six months right ventricular hypertrophy and dilatation were reduced. Because of the 2 documented cases of patients with PAH due to therapy with IFNα2 where PAH was reversible half a year after termination of IFNα2 [3,4] we attempted to terminate sildenafil under control of hemodynamic monitoring. PAP_mean _increased promptly to 57 mmHg. In this context we could verify the same effectiveness for therapy with vardenafil in comparison to sildenafil. Reduction of PVR was higher in vardenafil vs. sildenafil (Table 1); therefore the therapy was switched from sildenafil to vardenafil 10 mg twice daily. 24 months after onset of PAH the patient felt fine and resumed working. Vardenafil is still necessary to lower her PAP as demonstrated with transthoracic echocardiography: after pausing vardenafil for two days the PAP_syst _increased about 40 mmHg, but decreased again 120 minutes after administration of vardenafil. The heart diameter is steadily decreasing (right ventricular outflow tract from 39 to 27 mm and right atrium from 60–65 to 36–44 mm). Regarding the melanoma she remains relapse-free.

## Conclusion

IFN α2 is an accepted adjuvant treatment for patients with high risk melanoma [2]. Among the vascular complications reported for IFN are retinopathy [9], cutaneous vasculitis [10], gangrene requiring amputation and biopsy proven pulmonary vasculitis [3]. PAH includes various forms with different etiologies, but similar clinical presentation and functional derangement. Although PAH remains a rare disease, in recent times PAH related to other diseases has been better recognized. These forms are related to systemic connective tissue diseases, thromboembolic disease, congenital heart disease, portal hypertension, HIV infection, or are secondary to the use of drugs. They all result in an indistinguishable histological picture [11]. In addition to common hypertrophy of the tunica media, other proliferative lesions such as intimal thickening or plexiform lesions can be found. Moreover, in situ thrombosis and rarely isolated pulmonary arteritis can be observed in lungs of patients displaying PAH. Different pathomechanisms explaining these morphological changes of pulmonary vasculature have been discussed in the past, including endothelial and thrombocytic dysfunction, vasoconstriction, coagulation abnormalities, or cancer-like growth [12]. It is therefore of interest that IFNα can cause thrombotic microangiopathy which might contribute to the development of PAH [13].

Various cellular pathway abnormalities have been described that may play important roles in the development and progression of PAH [14,15]. These include altered synthesis of nitric oxide (NO), prostacyclin and endothelin, impaired potassium channel and growth factor receptor function, altered serotonin transporter regulation, increased oxidant stress, and enhanced matrix production [14-17]. However, the relative importance of each of these processes remains unclear. It has been reported, that in sheep IFNα is able to increase the PVR and the PAP by activation of the thromboxane-cascade with elevated levels of thromboxane-B2 in plasma and lung lymph [18]. Studies on the importance of inflammatory mediators, such as chemokines, in the lungs of PAH patients have led to a possible inflammatory component in the development of PAH [19]. This might be of relevance in IFN induced PAH since IFNα is known to induce expression of various chemokines [20].

All PAH result in similar histological remodelling of pulmonary arteries with thickening of the intima, proliferation of the media and plexogenic lesions. Today the pathophysiology of these lesions is much better understood and has resulted in new therapies involving substances such as prostacyclins, endothelin receptor antagonists or PDE-5 inhibitors, aimed not only at dilating arteries but also at preventing their remodelling.

Sildenafil and vardenafil inhibit PDE-5, an enzyme that is abundantly expressed in pulmonary vasculature [21]. PDE-5 is the major guanosine 3',5'-cyclic monophosphate (cGMP) degrading phosphodiesterase. PDE-5 gene expression and activity are increased in PAH [22]. Sildenafil binds to the catalytic site of PDE-5 approximately a thousand times more avidly than the natural substrate, cGMP [23]. cGMP is the second messenger of prostacyclin and NO and due to stabilization of this second messenger, therapy with PDE-5 inhibitors leads to prolongation of prostanoid- and NO-related vascular effects [24]. In patients with PAH, short-term application of sildenafil during right heart catheterization showed the potential to reduce PVR in a dose-dependent manner. Interestingly, the vasodilator effects were significantly stronger than with inhaled NO [25]. Advantages of a potential therapy with PDE-5-inhibitors for PAH are p.o. administration, an excellent safety profile and relatively low treatment costs [26]. A number of studies have shown the effectiveness of a therapy with PDE-5 inhibitors for PAH [25-32]. Because of the low incidence of this condition the number of patients studied is small. One study could show a long-lasting benefit with sildenafil with respect to hemodynamic and clinical parameters over 3, 6 and 12 months [26]. A recently published placebo-controlled and randomized phase III study revealed a significant reduction of PAP, improvement of 6-minute-walk test and reduction of hospitalization due to PAH [32].

Data are rare for the use of different PDE-5 inhibitors other than sildenafil in PAH. The in vitro biochemical potentials of sildenafil and vardenafil are comparable, whereas vardenafil has a binding affinity to PDE-5 more than ten times higher than sildenafil [33]. One could speculate that therapy with vardenafil in PAH could be more effective than with sildenafil due to its higher binding affinity to PDE-5. In a small study with different PDE-5 inhibitors in PAH, the reduction of PAP with sildenafil and vardenafil was comparable, while sildenafil showed surprisingly more selectivity for the pulmonary circulation and a better arterial oxygenation compared to vardenafil [34]. In the case presented our patient responded favorably to both sildenafil and vardenafil.

This is only the 5th case of IFN-induced PAH [3-5,7]. The previous patients received IFN for CML (three patients) or RCC (one patient). Our patient developed PAH after 2.5 years of adjuvant IFNα treatment, in the other cases the range was from several months up to 22 months after initiation of IFN therapy. In two cases PAH was reversible within 6 months after termination of IFNα therapy. In one case, the patient died 2 years after withdrawal from IFN therapy. An autopsy was not performed, but the authors suggested a systemic microangiopathy caused by IFNα as underlying reason [5]. To the best of our knowledge this is the first documented case where PAH was not reversible after termination of IFNα therapy and there was a need for continuous vasodilator therapy. Treatment with PDE-5-inhibitors had, in this case, a long-lasting beneficial effect. If IFNα treated patients develop respiratory symptoms, PAH should be considered in the differential diagnosis. For these patients PDE-5 inhibitors, e.g. sildenafil or vardenafil, could be an effective therapeutic approach.

## Abbreviations

cGMP = guanosine 3'5'-cyclic monophosphate

CML = chronic myelogenous leukemia

CT = computer tomopgraphy

IFN = Interferon

PAH = pulmonary arterial hypertension

PAP = pulmonary arterial pressure

PDE-5 = phosphodiesterase-5

PVR = pulmonary vasculature resistance

## Competing interest statement

The author(s) declare that they have no competing interests.

## Authors' contributions

NJ, ACB and GB treated the patient at the cardiology department. ACB, GB and SE performed the cardiological examinations. MAH, FK, WS and UT treated the patient at the dermatology department. NF, FK and UT drafted the manuscript. GB and WS revised the manuscript critically. All authors approved the final version of the manuscript.
